# Assessing Plasma Levels of Selenium, Copper, Iron and Zinc in Patients of Parkinson’s Disease

**DOI:** 10.1371/journal.pone.0083060

**Published:** 2013-12-10

**Authors:** Hai-Wen Zhao, Jie Lin, Xue-Bao Wang, Xing Cheng, Jian-Yong Wang, Bei-Lei Hu, Yan Zhang, Xiong Zhang, Jian-Hong Zhu

**Affiliations:** 1 Department of Preventive Medicine, Wenzhou Medical University, Wenzhou, Zhejiang, China; 2 Department of Geriatrics & Neurology, the Second Affiliated Hospital, Wenzhou Medical University, Wenzhou, Zhejiang, China; 3 Analytical and Testing Center, Wenzhou Medical University, Wenzhou, Zhejiang, China; 4 Key Laboratory of Nutrition and Metabolism, Institute for Nutritional Sciences, Chinese Academy of Sciences, Shanghai, China; 5 Key Laboratory of Systems Biology, Shanghai Institutes for Biological Sciences, Chinese Academy of Sciences, Shanghai, China; Florey Institute of Neuroscience and Mental Health , The University of Melbourne, Australia

## Abstract

Trace elements have been recognized to play an important role in the development of Parkinson’s disease (PD). However, it is difficult to precisely identify the relationship between these elements and the progression of PD because of an insufficient number of patients. In this study, quantifications of selenium (Se), copper (Cu), iron (Fe) and zinc (Zn) by atomic absorption spectrophotometry were performed in plasma from 238 PD patients and 302 controls recruited from eastern China, which is so far the largest cohort of PD patients and controls for measuring plasma levels of these elements. We found that plasma Se and Fe concentrations were significantly increased whereas Cu and Zn concentrations decreased in PD patients as compared with controls. Meanwhile, these four elements displayed differential changes with regard to age. Linear and logistic regression analyses revealed that both Fe and Zn were negatively correlated with age in PD patients. Association analysis suggests that lower plasma Se and Fe levels may reduce the risk for PD, whereas lower plasma Zn is probably a PD risk factor. Finally, a model was generated to predict PD patients based on the plasma concentrations of these four trace elements as well as other features such as sex and age, which achieved an accuracy of 80.97±1.34% using 10-fold cross-validation. In summary, our data provide new insights into the roles of Se, Cu, Fe and Zn in PD progression.

## Introduction

Parkinson’s disease (PD) is a chronic neurodegenerative movement disorder characterized by a progressive loss of dopaminergic neurons and formation of Lewy bodies in substantia nigra pars compacta. Origin of PD is rarely monogenic, and the majority are sporadic cases involving in multifaceted etiology including genetic factors and environmental exposures. Mitochondrial dysfunction, oxidative stress, abnormal protein accumulation, and neuroinflammation have been recognized as underlying causes in the pathogenesis of PD [1,2].

Neurochemical analyses have suggested a role for trace metals in PD, including selenium (Se), copper (Cu), iron (Fe) and zinc (Zn). For instance, α-synuclein, a major component of Lewy bodies, is able to interact with Fe, Cu and Zn leading to protein aggregation and crosslinking [3]. Amidst, Fe is the most extensively investigated element. Many studies have shown elevated Fe deposits in substantia nigra of PD patients, leading to oxidative stress and damage of nigrastriatal dopaminergic neurons [4,5]. Se, often considered as an antioxidant by incorporating into selenoproteins such as glutathione peroxidases and thioredoxin reductases, is also linked to PD [6]. Cu and Zn are essential elements for a variety of enzymes such as Cu/Zn superoxide dismutase and dopamine-beta hydroxylase, and have been suggested in the development of neurodegenerative processes due to their critical involvement in cellular regulation [7-9]. 

In past decades, alterations in Se, Cu, Fe and Zn levels have been investigated in plasma/serum, cerebral spinal fluid and substantia nigra of PD patients (Table S1). However, the number of patients is considerably limited in almost all studies, which could severely restrict the power of evaluation especially considering the high biological variability of trace element contents in the fluids [10]. Moreover, information concerning the relationship between these elements and PD largely remains mixed in plasma/serum. In this study, we recruited the largest cohort of PD patients and controls reported to date, and aimed to better understand the roles of Se, Cu, Fe and Zn in PD by assessing their levels in plasma. We investigated the interactions between each element as well as their relationships with age and clinical scores. We also analyzed element-element correlations as well as ratios in different ages and PD subtypes. Finally, we proposed a new model for prediction of PD patients. Our data provide new insights into understanding the alternations and potential roles of these four trace elements in PD patients.

## Materials and Methods

### Subjects

A total of 238 PD patients and 302 controls were enrolled in this case-control cohort study. The controls were selected to have similar age and gender distributions compared to those of the PD cohort (Table 1). The idiopathic PD patients were diagnosed according to the UK Parkinson’s Disease Society Brain Bank Criteria by two movement disorder specialists, and patients with a family history of PD, or with secondary and atypical Parkinsonism were excluded. All controls were free of neurological disorders by medical history, physical and laboratory examinations. Patients also underwent neurological examinations and were assessed with the Unified Parkinson Disease Rating Scale (UPDRS). Scores of UPDRS section II (UPDRS II), UPDRS section III (UPDRS III) and total UPDRS were 11.0±6.5, 24.9±15.0 and 38.1±21.8, respectively. Patients were divided into three subgroups: tremor-dominant, akinetic-rigid and mixed type (Table 1). The tremor and non-tremor scores for each patient were calculated as previously described [11]. All subjects were mainland Chinese ethnicity and gave written informed consent for the study under a protocol approved by the ethics board of the Second Affiliated Hospital, Wenzhou Medical University.

**Table 1:** Plasma trace elements concentration (µg/L) in PD patients and controls.

Category	Element	Controls			Patients			p value	Reference value
		mean±SD	n	age (mean±SD)	mean±SD	n	age (mean±SD)		mean±SD
Total**^*1*^**	Se*	105±33	302	65.6±12.2	115±37	238	66.6±11.3	0.005	111±22 [33]
	Cu*	1098±292			1014±274			0.001	1030±220 [26]
	Fe*	1470±648			1656±749			0.006	1440±770 [26]
	Zn*	1293±385			923±338			<0.001	1250±280 [34]
Male**^*2*^**	Se*	100±31	153	67.0±12.1	111±34	121	67.1±11.3	0.009	
	Cu*	1061±300			981±277			0.012	
	Fe*	1503±611			1736±814			0.016	
	Zn*	1289±375			921±341			<0.001	
Female**^*2*^**	Se	111±34	149	64.2±12.2	119±40	117	66.1±11.4	0.095	
	Cu*	1137±280			1049±267			0.017	
	Fe	1437±678			1574±661			0.077	
	Zn*	1298±396			924±337			<0.001	
Tremor-dominant**^*3*^**	Se				118±32	12	67.4±11.0	0.144	
	Cu				1083±219			0.771	
	Fe				1541±722			0.771	
	Zn*				929±419			0.004	
Akinetic-rigid**^*3*^**	Se				113±37	110	65.8±12.1	0.124	
	Cu*				985±268			0.001	
	Fe**^*#*^**				1524±726			0.713	
	Zn*				865±281			<0.001	
Mixed type**^*3*^**	Se*				117±38	116	67.3±10.5	0.010	
	Cu*				1034±282			0.049	
	Fe* **^*#*^**				1794±754			<0.001	
	Zn*				976±371			<0.001	

^*^ p <0.05 vs. their respective control subjects; PD, Parkinson’s disease.

^#^ p =0.057 between PD patients of akinetic-rigid and mixed type.

^1^ Compared between total patients and total controls.

^2^ Compared between patients and controls within the same gender.

^3^ Compared between clinical subtypes of PD patients and total controls.

### Chemicals

Ultrapure water was prepared by passing deionized water from a Milli-Q system (Millipore, Bedford, USA) and was used throughout the experiments. Nitric acid (65%) and hydrogen peroxide (30%) of spectroscopic grade (Merck, Darmstadt, Germany) were used for sample digestion and preparation of nitric acid stock standard solution (2%, v/v). All glassware was soaked in 10% of nitric acid (prepared from nitric acid of analytically pure, 65%, Juhua Group Corp., China) for 24h and rinsed with deionized water before use. Standard solutions of Se (100 µg/mL), Cu (500 µg/mL), Fe (1000 µg/mL) and Zn (500 µg/mL) were prepared by dilution of certiﬁed standard solutions (National Institute of Metrology, China). Matrix chemical modiﬁer for Se detection containing Pd(NO_3_)_2_ (2000 mg/L) and Mg(NO_3_)_2_ (1000 mg/L) were prepared from Pd(NO_3_)_2_ (10.0 g/L) for spectrum analysis (Merck, Darmstadt, Germany) and Mg(NO_3_)_2_ stock solution (2000 mg/L; from Mg(NO_3_)_2_·6H_2_O, Panreac, E.U.) using the nitric acid stock standard solution. Reference material Seronorm^TM^ Trace Elements Serum (Batch 201405, Sero AS, Norway) was used for accuracy control. 

### Sample collection and preparation

Fasting blood samples were collected in heparin tubes and centrifuged for 15 min at 1500 rpm at room temperature. After centrifugation, plasma samples were collected and stored at -80°C until analysis. Hemolyzed samples were excluded from the study. Utmost care was taken to avoid potential pre-analytical contamination during specimen acquisition and treatment. A conventional wet acid digestion method was used to digest plasma as previously described with slight modification [12]. Briefly, 0.4 mL of plasma sample and 3 mL of nitric acid (65%) were added into a 25 mL beaker, and digested at 70-80°C on an electric hot plate for 90 min. Thereafter, 1.5 mL of hydrogen peroxide (30%) was added into the beaker and continued for digestion. When the remaining volume was about 0.5-1 mL, the liquid was all transferred into a 5 mL of volumetric flask after cooling, and a final volume of 5 mL was obtained by adding the nitric acid stock standard solution. The reference material was treated in the same manner before analysis. 

### Element analyses

Zeeman atomic absorption spectroscopy (SpectrAA240Z; Varian, USA) equipped with graphite tube atomizer (GTA120; Varian, USA) was used for determination of plasma Se and Cu concentrations, and fast sequential atomic absorption spectroscopy (SpectrAA240FS; Varian, USA) equipped with deuterium background correction was used for determination of plasma Fe and Zn concentrations. The hollow cathode lamps of Se, Cu, Fe and Zn were run under the conditions suggested by the manufacturer. A single element hollow cathode lamp was operated at 10.0 mA for selenium, 4.0 mA for copper, and 5.0 mA for Fe and Zn, with a spectral bandwidth of 1.0 nm for Se and Cu, and 0.2 nm for Fe and Zn. The analytical wavelengths were set at 196.0, 324.8, 248.3, and 213.9 nm for Se, Cu, Fe and Zn, respectively. The graphite tube heating programs optimized for analyses of Se and Cu were showed in Table S2. A standard addition technique was applied for Se quantification, and the standard addition concentration was 25 µg/L. The injection volume was 25 µL for Se (6 µL of matrix modifier, 4 µL of standard addition solution, and 15 µL of sample) and 12 µL for Cu. For Fe and Zn, the flow rate was 13.5 L/min for air (oxidant) and 2.0 L/min for acetylene, and the time of delay and reading was 7 sec each.

### Statistical analysis

Statistical analysis was performed by using SPSS (version 18.0) for windows. First, we used Kolmogorov-Smirnov (KS) test and Shapiro-Wilk (W) test to tell if our data come from a Gaussian population, and both results suggested that the distribution of our data set could not be well-modeled by normal distribution. We then tried to transform our data towards normality by using different statistical methods including BoxCox analysis, natural or base-10 log transformation as well as square root transformation; however, none of them was successful. Thus, non-parametric analysis was adopted in this study. Mann-Whitney U-test was used to examine the significance of difference between two groups. Linear and logistic regression models were used to observe the relationship between element and age. Subjects were then divided into four groups based on the quartiles of element concentrations with the highest one as the reference group. Logistic regression was used to calculate the odds ratio (OR) and 95% confidence interval (CI) for the association between PD and each quartile of plasma trace elements. Model 1 was for individual trace element adjusted with age and sex, while Model 2 was for all four elements as ordinal variables adjusted with age and sex. Trend analyses across quartiles were performed using the ordinal variables containing median element concentrations for each quartile. The correlation of two examined elements or between element and UPDRS was detected by Spearman correlation analysis. Difference was considered significant if p value (FDR corrected) <0.05. 

### A model for PD prediction

The support vector machine (SVM) algorithm, which is a powerful supervised machine learning model for classification and regression analysis [13], was used to build a model for PD prediction. Given a set of training samples with plasma concentrations of Fe, Zn, Cu and Se, sex and age compositions as well as their known categories (in our model, 0 and 1 represent controls and patients, respectively), a SVM model was generated using the R (version 2.14.0) e1071 package (version 1.6.1) [14]. 10-fold cross-validation was used to train and test the SVM classifier to get an optimal performance. First, we randomly split our data set into 10 equal parts. Each of the 10 parts was then used as a testing set while the remaining 9 parts as a training set. For each time, a PD model was generated by training from the information involving element concentrations, sex and age compositions of the training set and was used to assign the testing set to get prediction categories. Thus, a total of 10 models were obtained based on one 10-fold cross-validation. The receiver operating characteristic (ROC) curve which was generated by the R package ROCR (version 1.0.4) [15] was used to evaluate the performance of each model. This process was repeated for 100 times to assess the average performance of our PD model. 

## Results

### General analysis of plasma trace element concentrations in PD patients

The measurement of element concentrations was initially assessed by analyzing reference material Seronorm^TM^ Trace Elements Serum. The results showed that the accuracy ranged from 99% for Zn to 107% for Fe. The precision of the method varied from 1.1% for Zn to 5.1% for Se. In addition, the detection limits, calculated as three times the blank intensity SD, were 1.65 µg/L for Se, 0.24 µg/L for Cu, 31.44 µg/L for Fe and 5.00 µg/L for Zn, respectively. 

**Figure 1:**
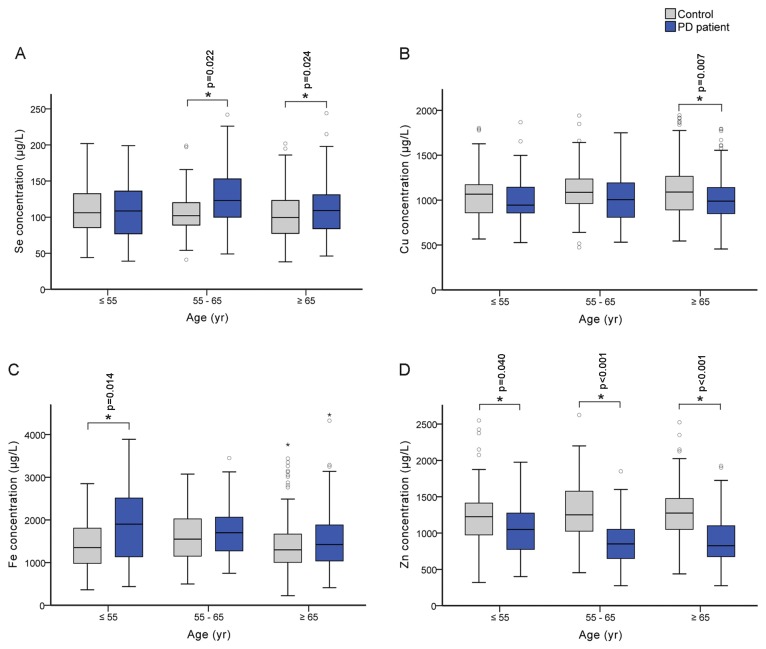
Plasma trace element concentrations in three age groups of PD patients and controls. The number of subjects with age ≥55, 55~65, ≤65 were 38, 53, 147 respectively for PD patients, and 71, 59, 172 respectively for controls. Box charts represent the distribution of plasma Se (A), Cu (B), Fe (C) and Zn (D) concentrations. Data were analyzed with Mann-Whitney U-test and significant difference was marked with * for p <0.05.

Our quantifications in plasma showed that Se and Fe concentrations were significantly increased by 9.5% (p=0.005) and 12.7% (p=0.006), respectively, while Cu and Zn levels were decreased by 7.7% (p=0.001) and 28.6% (p<0.001), respectively in PD patients (Table 1). Similar trends of changes were also observed in the male and female subgroups in spite that no significant change for Se and Fe levels was observed in the female patients. To further investigate the influence of age, we divided all the subjects into three age groups (≤55, 55~65 and ≥65; Figure 1). Interestingly, only plasma Zn level in PD patients was significantly decreased regardless of age status (Figure 1D), suggesting that reduced plasma Zn concentration might be a potential signal for PD early warning. The other three examined elements only had significant changes in certain age groups. For example, Se level was found significantly increased in PD patients with age >55 (55~65 and ≥65 groups), Fe level increased only in patients with age ≤55, whereas Cu level decreased in PD patients with age ≥65 (Figure 1). Therefore, age could be an important factor that affects the absorption or metabolism of these trace elements in PD patients. When compared plasma level between three clinical subtypes of PD (tremor-dominant, akinetic-rigid and mixed types) and total controls, we found plasma Zn significantly decreased in all three clinical subtypes and Cu significant decreased in akinetic-rigid and mixed types, whereas Se and Fe significantly increased only in mixed types (Table 1). We also compared plasma element levels between different clinical subtypes of PD. Only plasma Fe level in akinetic-rigid patients appeared lower than that in the mixed type (p=0.057). 

**Figure 2:**
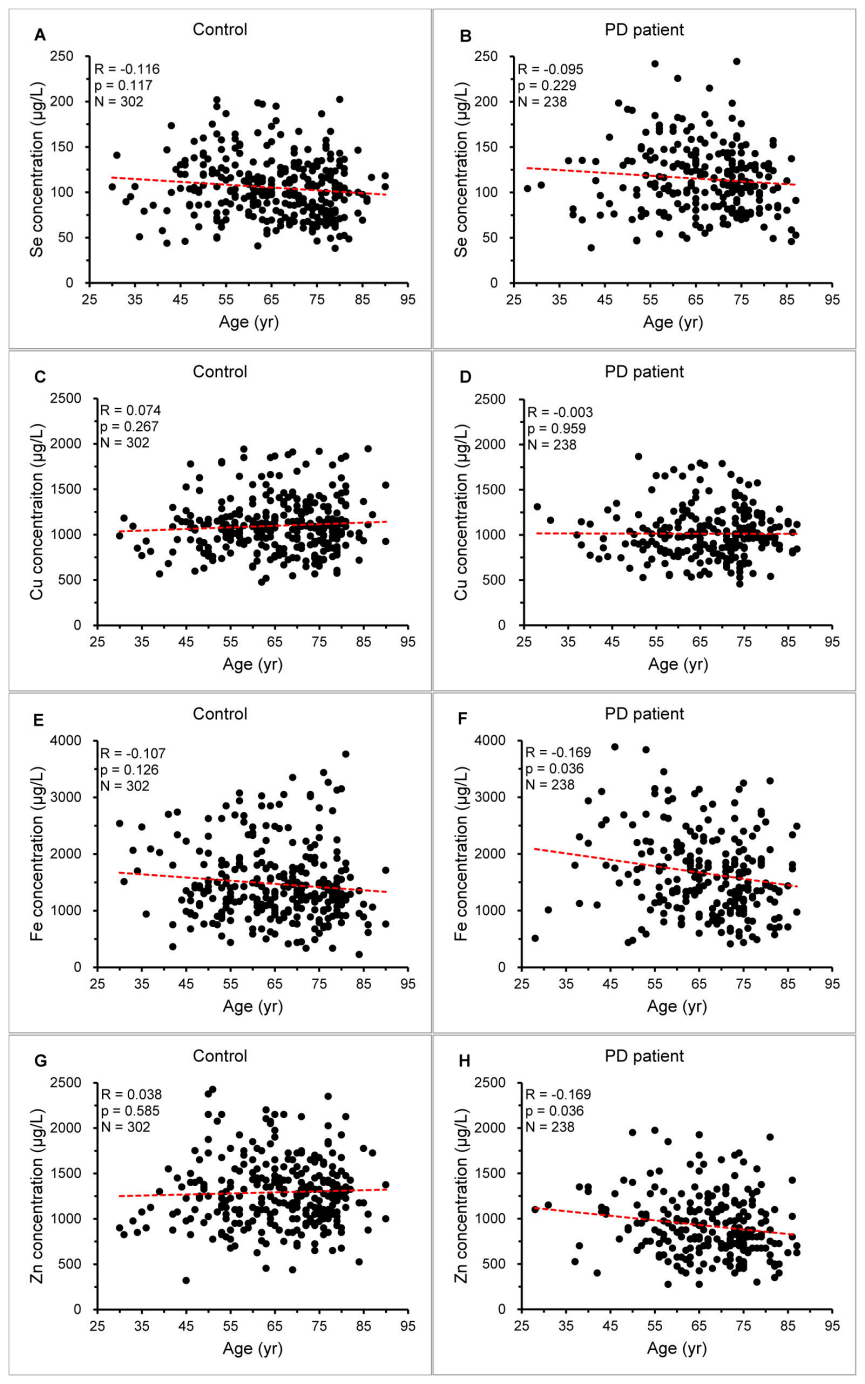
Relationship between plasma trace element levels and age. The scatters represent the correlation of plasma Se (A and B), Cu (C and D), Fe (E and F) and Zn (G and H) levels with age in PD patients and controls. The parameters showed in the figure represent the performance of the linear regression (red dash line).

Linear regression model was used to analyze the relationship between element level and age in both controls and PD subjects (Figure 2). With the increase of age, only Fe and Zn concentrations were linearly decreased in PD patients (p=0.036 for both elements; Figure 2F and H). Logistic regression model was also used and the significance results were quite similar to those using the linear model (Table S3). Logistic regression analysis was carried out to further explore the role of these elements in PD. In the analyses of individual plasma trace element (Model 1), we found Zn was associated with PD of age at 55~65 (p for trend =0.004; Table S4) and Se was associated with PD of age ≥65 (p for trend =0.024; Table S4). When together considering all four plasma trace elements (Model 2), both Zn and Fe, potentially Se, were associated with PD in total subjects (p for trend =0.040 for Zn and Fe, and =0.055 for Se; Table 2), and Zn was also associated with PD in the group of age at 55~65 (p for trend =0.044; Table S4). In either Model 1 or 2, lower plasma Se and Fe levels appeared to be protective against PD, while lower plasma Zn level in particular led to increased risk for PD (Table 2 and S4). 

**Table 2:** Association analysis of plasma trace elements with PD^1^.

Element	Category	Nagelkerke R square	First quartile	Second quartile	Third quartile	Fourth quartile	p value for trend**^*2*^**
Se	Concentration	-	<83	83-106	107-130.75	>130.75	-
	n (control/PD)	-	80/53	89/53	73/57	60/75	-
	Model 1	0.031	0.505 (0.308,0.827)	0.461 (0.284,0.749)	0.598 (0.366,0.977)	1	0.153
	Model 2	0.396	0.218 (0.114,0.415)	0.252 (0.137,0.462)	0.431 (0.234,0.793)	1	0.055
Cu	Concentration	-	<862	862-1038	1039-1191.5	>1191.5	-
	n (control/PD)	-	62/72	67/69	88/47	85/50	-
	Model 1	0.041	2.065 (1.259,3.386)	1.782 (1.093,2.905)	0.922 (0.560,1.518)	1	0.153
	Model 2	0.396	1.560 (0.842,2.892)	1.336 (0.737,2.421)	0.830 (0.454,1.516)	1	0.250
Fe	Concentration	-	<1063	1063-1413	1414-1888	>1888	-
	n (control/PD)	-	80/53	88/52	73/61	61/72	-
	Model 1	0.029	0.529 (0.323,0.868)	0.471 (0.288,0.769)	0.681 (0.419,1.107)	1	0.150
	Model 2	0.396	0.348 (0.189,0.641)	0.386 (0.214,0.698)	0.509 (0.286,0.907)	1	0.040
Zn	Concentration	-	<825	825-1100	1101-1375	>1375	-
	n (control/PD)	-	21/103	84/77	90/37	107/21	-
	Model 1	0.302	24.875 (12.806,48.320)	4.685 (2.673,8.211)	2.093 (1.143,3.834)	1	0.128
	Model 2	0.396	40.728 (19.327,85.828)	7.067 (3.766,13.264)	2.698 (1.401,5.195)	1	0.040

^1^ Odds ratios (95% CI) from logistic models were represented for dichoutomous outcomes (presence of PD) with the highest quartile as the reference group. Model 1 was for individual trace element adjusted with age and sex; Model 2 was for all four elements as ordinal variables adjusted with age and sex.

^2^ Trend analyses across quartiles were performed using the ordinal variables containing median element concentrations for each quartile.

### Correlation analysis of trace elements

We used the Spearman’s correlation coefficient (SCC) to investigate the correlation of any two examined elements between PD patients and controls (Table 3). Although some element pairs showed certain correlations, no obvious correlation (SCC>0.6 as defined here) could be found among them except for Fe~Zn in tremor-dominant subtypes of PD patients (SSC=0.958, p=0.010; Table 3). Similarly, no obvious correlation could be detected between elements and UPDRS scores in either all patients or in different gender, age or clinical subgroups (Table S5). This implies that element concentrations may be relatively independent from each other and have little influence on UPDRS, at least in our samples.

**Table 3:** Spearman correlation coefficients between plasma trace element concentrations.

Subjects	Category (n)	Se-Cu	Se-Fe	Se-Zn	Cu-Fe	Cu-Zn	Fe-Zn
Control	Total (302)	**0.235**	0.064	**0.332**	-0.097	**0.178**	0.098
	Male (151)	0.176	0.063	**0.319**	-0.173	0.091	0.120
	Female (149)	**0.235**	0.088	**0.341**	-0.008	**0.257**	0.086
	Age ≤55 (71)	**0.309**	0.127	**0.339**	-0.147	0.067	0.075
	Age 55~65 (59)	0.045	0.230	**0.386**	-0.032	0.278	-0.023
	Age ≥65 (172)	**0.268**	-0.016	**0.321**	-0.098	**0.184**	**0.164**
Patient	Total (238)	**0.332**	0.147	**0.245**	0.024	**0.285**	**0.384**
	Male (121)	0.237	0.156	0.150	0.115	**0.269**	**0.382**
	Female (117)	**0.414**	0.151	**0.325**	-0.035	**0.328**	**0.395**
	Age ≤55 (38)	0.306	<0.001	0.456	-0.034	0.484	0.308
	Age 55~65 (53)	0.348	0.279	0.373	0.009	0.308	**0.457**
	Age ≥65 (147)	**0.332**	0.141	0.183	0.037	**0.244**	**0.383**
	tremor-dominant (12)	0.460	0.589	0.582	0.210	0.126	**0.958**
	akinetic-rigid (110)	**0.262**	0.089	0.201	-0.003	**0.410**	**0.307**
	mixed type (116)	**0.416**	0.177	**0.254**	0.010	0.163	**0.363**

Bold values are significant for p value <0.05.

**Figure 3:**
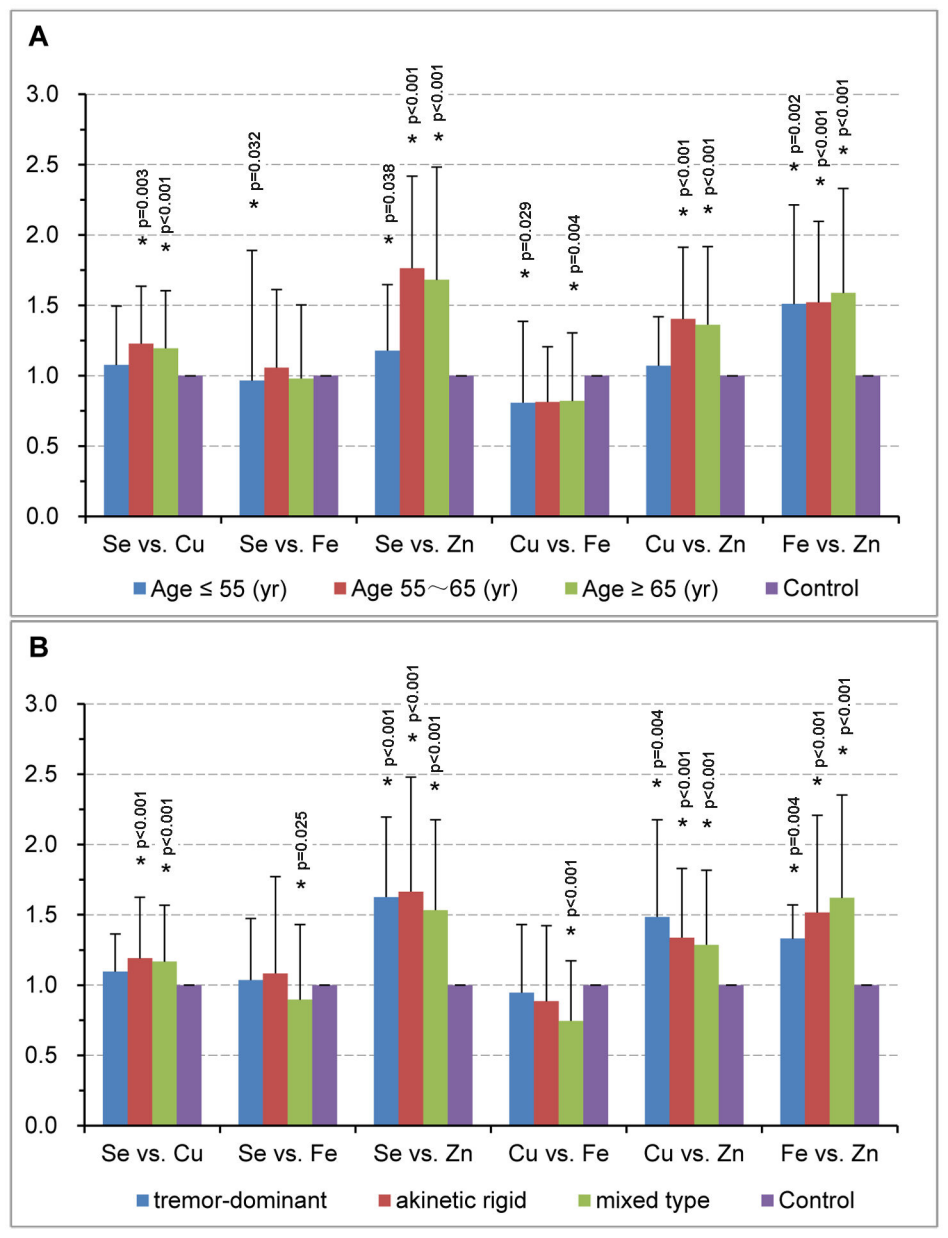
Element-element ratios for age groups and clinical subtypes. Element-element ratios for three age groups in PD patients (age ≤55, 55~65 and ≥65) were compared with their respective age-matched controls (A), and for clinical subtypes in PD patients were compared with total controls (B). Histograms represent the fold changes of each element-element ratio compared to the mean value of their respecitve controls which is set as 1. Data were analyzed with Mann-Whitney U-test and significant difference was marked with * for p <0.05.

We also explored ratios of any two element levels in different age groups and clinical subtypes. Compared with controls, significantly increased Se/Zn, Cu/Zn and Fe/Zn ratios were detected in all three age groups and all subtypes of PD except for Cu/Zn at age ≤55 (Figure 3). Similar trends for Cu/Zn and Fe/Zn have been observed previously in PD subtypes [16]. We further analyzed each PD subtypes based on different age ranges. Interestingly, these three ratios (Se/Zn, Cu/Zn and Fe/Zn) had significant changes in all subtypes at age ≥65 as well as in akinetic-rigid and mixed types at age of 55~65, and Fe/Zn also showed a marked increase in the mixed type at age ≤55 (Figure S1). With regard to other element ratios, their changes varied in different subtypes. For example, a significant decrease of Se/Fe ratio was observed only in the mixed type (Figure 3B). Further analysis of different age ranges in the mixed type revealed that Se/Fe ratio was solely significantly decreased in patients at age ≤55 (Figure S1). These results suggest that element ratios could be affected by both age and PD subtypes.

### A SVM model for PD prediction

**Figure 4:**
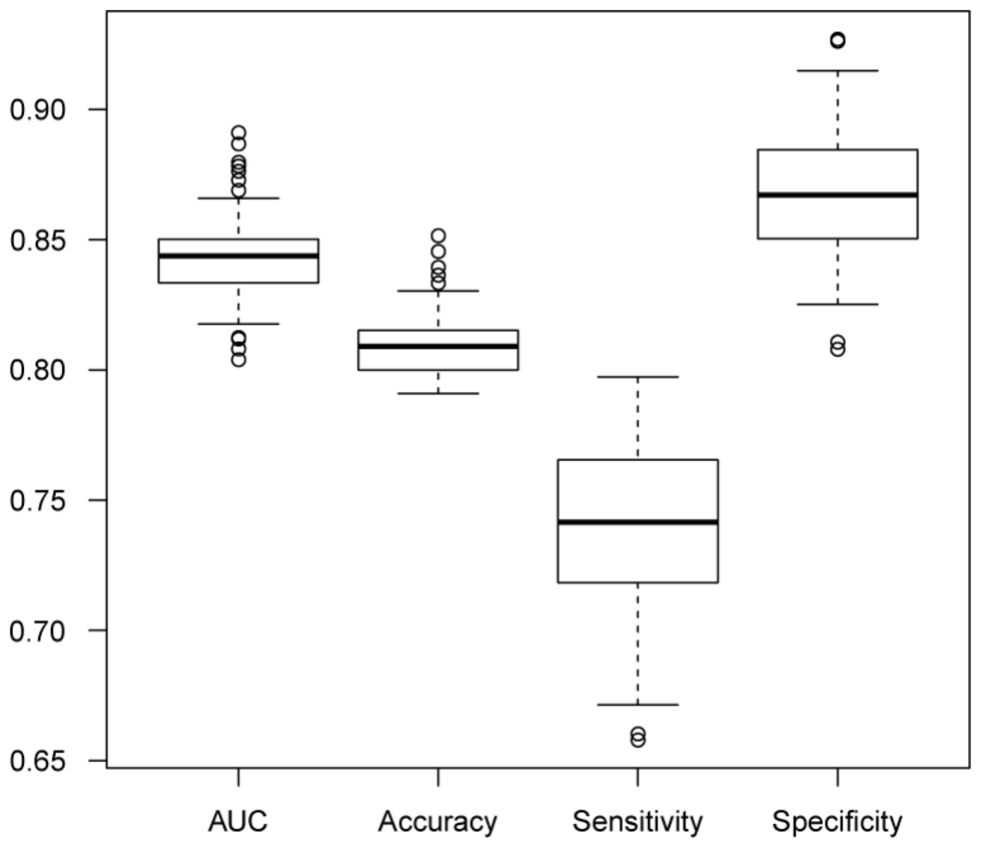
Performance of 10-fold cross-validation SVM model. The generalized performance of the SVM model. We rebuilt the model for 100 times for the validation using AUC, accuracy, sensitivity and specificity.

A PD model which could be used to predict PD patients was built based on the plasma trace element concentrations and other information. Several algorithms had been tried including clustering, decision tree analysis, conditional inference tree analysis, random forest, and SVM, which displayed an accuracy rate at <50%, 72.03%, 78.26%, 74.34%, and 80.97%, respectively. Among them, SVM showed the best performance. After 10-fold cross-validation to build an optimal machine learning model and 100 repeats of this process, our model achieved a prediction accuracy of 80.97±1.34% , the area under the ROC curve (AUC) of 84.32±1.55%, a sensitivity of 74.09±3.14%, and a specificity of 86.78±2.55%, respectively. The validation of these metrics revealed that our model has a quite stable performance for PD prediction (Figure 4). 

Considering that the plasma concentrations of some elements were related to ages, it is possible that the prediction performance of our model might be age-related. After rebuilding the SVM model within each of the three age groups defined above, the result showed that, regardless the same or actual size of each cohort, the model achieved a better performance with age >55 (≥65 and 55~65 groups; Figure S2). This result implied that our model might be more suitable for PD prediction in elder age ranges. In the future, the performance of this model should be improved if more samples and/or plasma concentrations of additional trace elements are available.

## Discussion

Pathogenesis of PD is a complex process involving multiple genetic and environmental factors, where role of elements has been investigated in a number of studies [17,18]. However, the changes of trace elements mostly remain elusive in PD, probably due to the limited number of observations and population variations. In this study, we assessed plasma levels of Se, Cu, Fe and Zn in 238 PD patients and 302 controls with similar age and gender distribution in eastern China, representing the largest cohort thus far in investigating PD-associated changes in plasma elements.

Se is involved in anti-oxidative defense as a key element in multiple enzymes/proteins. Unexpectedly, our data presented an increased Se level in plasma of PD patients, and suggest Se as a potential risk factor for PD. On the other hand, oxidative stress conferred in PD pathogenesis may lead to a higher presence of Se level to raise antioxidant ability as a protective mechanism. Further analysis showed Se level was found increased only in the mixed type of PD patients, indicating Se as an antioxidant may be more demanding in this subtype. Shahar et al recently showed in a long-term study that plasma Se was not associated with the presence of PD but positively related to performance in neurological tasks assessing coordination and motor speed [19]. Interestingly, our results indicate Se was potentially associated with PD, particularly in older population (age ≥65), but we did not find an obvious correlation between plasma Se concentration and UPDRS III, an evaluation of motor activity in PD. The discrepancy is probably due to large differences in experiment designs and assessment of motor activities. 

Increased Fe deposits have been consistently found in substantia nigra of PD patients [20-22]. However, information is a bit mixed concerning Fe level in plasma/serum of PD patients, which showed all directions of changes from a decrease [16,23], to no change [10,24,25], and to an increase [26]. In the present study, we found plasma Fe level was only elevated in younger (age ≤55) PD patients and decreased ever since, which somewhat explains the dissimilarity of plasma/serum Fe levels in previous reports when only recruiting limited sample size. More importantly, the above data suggest an early rise of plasma Fe in PD development, likely leading to the increased Fe deposit in substantia nigra and then contributing to the pathogenesis of PD. The latter was in accordance with our further analysis which indicates a potential association between increased Fe level and aggravated risk for PD. Together, our data provided evidence supporting the pathological role of Fe in PD. The differential presence of plasma Fe concentrations in the akinetic rigid and mixed subtypes suggests that Fe may also have an impact on clinical manifestations of PD. 

Although a reduction of Cu level was found in plasma of PD patients, our further analyses including with clinical subtypes/scores, risk association, age correlation and with other elements revealed a weak participation of Cu in PD development and progression. In fact, plasma Cu reduction was present only in older PD patients (age ≥65), indicating Cu change may merely be a consequence of PD. Despite a high level of heterogeneity, Mariani et al. recently presented a meta-analysis showing plasma/serum Cu and Fe did not differ much in PD [27]. Based on our results, age may serve an important factor when analyzing changes of Cu and Fe in PD, which unfortunately was missed in their study. 

On the other hand, plasma Zn showed the most striking change showing a substantial drop in PD patients. The interaction between plasma Zn and age also showed a drastic transition, from a slight positive correlation in controls to a significant negative correlation in PD affected subjects, suggesting an increasing or accumulative loss of Zn in PD progression. Indeed, similar decreases of Zn by 27.1% and 23.1% in plasma/serum of PD patients have been previously reported in small populations [16,28], whereas a few other studies showed no change of plasma/serum Zn level [10,26,29]. Although population variation and high biological variability in fluids may likely be counted as major reasons, it is still a bit surprising such a difference did not appear in most cohorts of quantifications. In fact, evidence of functional Zn deficiency in PD has been previously noted by oral Zn tally test and measurements of three Zn status-related variables [8]. Our further analysis showed lower plasma Zn level was linked with increased risk for PD, suggesting Zn addition might be helpful in PD treatment. Consistently, in a Drosophila PD model, Zn supplementation greatly improved PD phenotypes induced by expression of parkin mutant [30]. Further investigation is therefore warranted to clarify the role of Zn in the development and treatment of this neurodegenerative disorder.

 We failed to detect any obvious correlation between element pairs except for Fe-Zn in the tremor-dominant type of PD patients, neither between elements and UPDRS scores, while the latter is in line with previous studies [29,31,32], implying that element levels may be relatively independent from each other and have little impact on clinical scores. However, further analysis of element-element ratios revealed that the significant ratio changes involving Zn occurred in nearly all different ages and PD subtypes, while other ratio changes varied in ages and subtypes when compared to the control. Such an involvement of Zn could be simply due to a more dramatic alternation in plasma Zn than in the other elements, while if not, may reflect a previously unrecognized position of Zn in the network of trace elements. 

Another contribution of this study is to build a SVM-based model for the prediction of PD mainly based on the four element concentrations. The ROC curve and validation test showed a good performance for this model. Further tests for different age groups suggest that the SVM model might have a better fitness for elder age ranges. To our knowledge, this is the first time to use four plasma trace element concentrations to build a PD model with decent outputs. Further efforts are needed to use new algorithms or more information to improve this model for a better performance.

## Conclusions

In conclusion, our study demonstrated increased levels of Se and Fe, and decreased levels of Cu and Zn in plasma of PD patients, and presented detailed information of their roles with regard to age and clinical scores/subtypes. Plasma Se or Fe elevation resulted in increased risk towards PD while plasma Zn appeared to be a protective factor against PD. We also built a SVM-based model to predict PD mainly based on these four trace elements. Our data provide novel understanding of plasma Se, Cu, Fe and Zn in PD pathogenesis and progression.

## Supporting Information

Table S1 **Published changes of Se, Cu, Fe and Zn levels in PD.**
(DOC)

Table S2 **The graphite tube heating programs optimized for analyses of Se and Cu.**
(DOC)

Table S3 **Comparison of linear regression model and logistic regression model for the relationship between plasma element level and age in both PD and control subjects.**
(DOC)

Table S4 **Association analysis of plasma trace elements with PD by gender or age group.**
(DOC)

Table S5 **Spearman correlation coefficients between plasma element concentrations and UPDRS scores in PD patients.**
(DOC)

Figure S1 **Element-element ratios for clinical subtypes based on age.** Element-element ratio for each clinical subtype in PD patients was compared with age-matched controls of age ≤55 (A), 55~65 (B) and ≥65 (C). Histograms represent the fold changes of each element-element ratio compared to the mean value of their respecitve age-matched controls which was set as 1. Data were analyzed with Mann-Whitney U-test and significant difference was marked with * for p <0.05.
(TIF)

Figure S2 **Generalized performance for SVM model based on ages.** 10-fold cross-validation SVM model for PD prediction was built for 100 times based on age ≤55 (A), 55~65 (B) and ≥65 (C).
(TIF)
