# Supplementary material for: Assessing Plasma Levels of Selenium, Copper, Iron and Zinc in Patients of Parkinson’s Disease
Source: PLoS One. 2013 Dec 10;8(12):e83060. doi: 10.1371/journal.pone.0083060 (PMC3858355; doi:10.1371/journal.pone.0083060)
Supplement: Table S1 — Published changes of Se, Cu, Fe and Zn levels in PD. (DOC) [file pone.0083060.s001.doc]

**Table S1** Published changes of Se, Cu, Fe and Zn levels in PD

| Element | Plasma/serum | | | Cerebral spinal fluid | | | Substantia nigra | | |
| --- | --- | --- | --- | --- | --- | --- | --- | --- | --- |
|  | Change | Number | Ref | Change | Number | Ref | Change | Number | Ref |
| Se | ↑n.s. | 28/43 |  | ↑n.s. | 28/43 |  |  |  |  |
|  | ↑ | 36/21 |  | ↑ | 36/21 |  |  |  |  |
|  | ↓ | 40/40 |  |  |  |  |  |  |  |
|  | ↓ | 45/42 |  |  |  |  |  |  |  |
| Cu | – | 39/39 |  | ↑ | 24/34 |  | ↓ | 8/3 |  |
|  | – | 37/37 |  | – | 11/22 |  | ↓n.s. | 14/7 |  |
|  | – | 40/40 |  | – | 37/37 |  | - | 17/29 |  |
|  | ↓ | 26/13 |  | – | 49/26 |  |  |  |  |
|  | – | 36/21 |  | – | 26/13 |  |  |  |  |
|  | ↓ | 64/28 |  | – | 36/21 |  |  |  |  |
|  | ↓ | 40/40 |  | ↑ | 22/- |  |  |  |  |
|  | ↑ | 45/42 |  | ↑ | 20/15 |  |  |  |  |
|  | – | 41/26 |  |  |  |  |  |  |  |
|  | – | 82/82 |  |  |  |  |  |  |  |
|  | – | 22/49 |  |  |  |  |  |  |  |
| Fe | – | 68/68 |  | – | 26/33 |  | ↑ | 8/3 |  |
|  | ↓ | 104/352 |  | – | 11/22 |  | ↑ | 8/7 |  |
|  | – | 37/37 |  | – | 37/37 |  | ↑ | 14/16 |  |
|  | – | 30/30 |  | – | 26/13 |  | - | 14/16 |  |
|  | – | 26/13 |  | ↑ | 36/21 |  | ↑ | 6/6 |  |
|  | – | 40/29 |  | – | 20/15 |  | ↑ | 16/14 |  |
|  | – | 36/21 |  |  |  |  | ↑ | 1/1 |  |
|  | ↓ | 45/42 |  |  |  |  | - | 17/29 |  |
|  | – | 41/26 |  |  |  |  | ↑ | 40/26 |  |
|  | ↑ | 82/82 |  |  |  |  | ↑ | 9/11 |  |
|  | – | 22/49 |  |  |  |  |  |  |  |
| Zn | – | 32/39 |  | ↓ | 37/37 |  | ↑ | 8/3 |  |
|  | – | 37/37 |  | – | 26/13 |  | – | 14/16 |  |
|  | – | 26/13 |  | – | 36/21 |  | ↓ | 1/1 |  |
|  | – | 36/21 |  | ↑ | 20/15 |  |  |  |  |
|  | ↓ | 40/40 |  |  |  |  |  |  |  |
|  | ↓ | 45/42 |  |  |  |  |  |  |  |
|  | – | 82/82 |  |  |  |  |  |  |  |

↑, increased; ↓, decreased; –, not changed; n.s., not significant; Number, patients/controls; Ref, reference

**Reference**

1. Aguilar MV, Jimenez-Jimenez FJ, Molina JA, Meseguer I, Mateos-Vega CJ, et al. (1998) Cerebrospinal fluid selenium and chromium levels in patients with Parkinson's disease. J Neural Transm 105: 1245-1251.

2. Qureshi GA, Qureshi AA, Memon SA, Parvez SH (2006) Impact of selenium, iron, copper and zinc in on/off Parkinson's patients on L-dopa therapy. J Neural Transm Suppl: 229-236.

3. Nikam S, Nikam P, Ahaley SK, Sontakke AV (2009) Oxidative stress in Parkinson's disease. Indian J Clin Biochem 24: 98-101.

4. Ahmed SS, Santosh W (2010) Metallomic profiling and linkage map analysis of early Parkinson's disease: a new insight to aluminum marker for the possible diagnosis. PLoS One 5: e11252.

5. Jimenez-Jimenez FJ, Fernandez-Calle P, Martinez-Vanaclocha M, Herrero E, Molina JA, et al. (1992) Serum levels of zinc and copper in patients with Parkinson's disease. J Neurol Sci 112: 30-33.

6. Pall HS, Williams AC, Blake DR, Lunec J, Gutteridge JM, et al. (1987) Raised cerebrospinal-fluid copper concentration in Parkinson's disease. Lancet 2: 238-241.

7. Dexter DT, Carayon A, Javoy-Agid F, Agid Y, Wells FR, et al. (1991) Alterations in the levels of iron, ferritin and other trace metals in Parkinson's disease and other neurodegenerative diseases affecting the basal ganglia. Brain 114 ( Pt 4): 1953-1975.

8. Jimenez-Jimenez FJ, Molina JA, Aguilar MV, Meseguer I, Mateos-Vega CJ, et al. (1998) Cerebrospinal fluid levels of transition metals in patients with Parkinson's disease. J Neural Transm 105: 497-505.

9. Gazzaniga GC, Ferraro B, Camerlingo M, Casto L, Viscardi M, et al. (1992) A case control study of CSF copper, iron and manganese in Parkinson disease. Ital J Neurol Sci 13: 239-243.

10. Loeffler DA, LeWitt PA, Juneau PL, Sima AA, Nguyen HU, et al. (1996) Increased regional brain concentrations of ceruloplasmin in neurodegenerative disorders. Brain Res 738: 265-274.

11. Torsdottir G, Kristinsson J, Sveinbjornsdottir S, Snaedal J, Johannesson T (1999) Copper, ceruloplasmin, superoxide dismutase and iron parameters in Parkinson's disease. Pharmacol Toxicol 85: 239-243.

12. Wypijewska A, Galazka-Friedman J, Bauminger ER, Wszolek ZK, Schweitzer KJ, et al. (2010) Iron and reactive oxygen species activity in parkinsonian substantia nigra. Parkinsonism Relat Disord 16: 329-333.

13. Forte G, Bocca B, Senofonte O, Petrucci F, Brusa L, et al. (2004) Trace and major elements in whole blood, serum, cerebrospinal fluid and urine of patients with Parkinson's disease. J Neural Transm 111: 1031-1040.

14. Boll MC, Sotelo J, Otero E, Alcaraz-Zubeldia M, Rios C (1999) Reduced ferroxidase activity in the cerebrospinal fluid from patients with Parkinson's disease. Neurosci Lett 265: 155-158.

15. Bharucha KJ, Friedman JK, Vincent AS, Ross ED (2008) Lower serum ceruloplasmin levels correlate with younger age of onset in Parkinson's disease. J Neurol 255: 1957-1962.

16. Boll MC, Alcaraz-Zubeldia M, Montes S, Rios C (2008) Free copper, ferroxidase and SOD1 activities, lipid peroxidation and NO(x) content in the CSF. A different marker profile in four neurodegenerative diseases. Neurochem Res 33: 1717-1723.

17. Hozumi I, Hasegawa T, Honda A, Ozawa K, Hayashi Y, et al. (2011) Patterns of levels of biological metals in CSF differ among neurodegenerative diseases. J Neurol Sci 303: 95-99.

18. Ling H, Bhidayasiri R (2011) Reduced serum caeruloplasmin levels in non-wilsonian movement disorders. Eur Neurol 66: 123-127.

19. Fukushima T, Tan X, Luo Y, Kanda H (2011) Serum vitamins and heavy metals in blood and urine, and the correlations among them in Parkinson's disease patients in China. Neuroepidemiology 36: 240-244.

20. Mariani S, Ventriglia M, Simonelli I, Donno S, Bucossi S, et al. (2013) Fe and Cu do not differ in Parkinson's disease: a replication study plus meta-analysis. Neurobiol Aging 34: 632-633.

21. Cabrera-Valdivia F, Jimenez-Jimenez FJ, Molina JA, Fernandez-Calle P, Vazquez A, et al. (1994) Peripheral iron metabolism in patients with Parkinson's disease. J Neurol Sci 125: 82-86.

22. Logroscino G, Marder K, Graziano J, Freyer G, Slavkovich V, et al. (1997) Altered systemic iron metabolism in Parkinson's disease. Neurology 49: 714-717.

23. Sofic E, Paulus W, Jellinger K, Riederer P, Youdim MB (1991) Selective increase of iron in substantia nigra zona compacta of parkinsonian brains. J Neurochem 56: 978-982.

24. Mann VM, Cooper JM, Daniel SE, Srai K, Jenner P, et al. (1994) Complex I, iron, and ferritin in Parkinson's disease substantia nigra. Ann Neurol 36: 876-881.

25. Loeffler DA, Connor JR, Juneau PL, Snyder BS, Kanaley L, et al. (1995) Transferrin and iron in normal, Alzheimer's disease, and Parkinson's disease brain regions. J Neurochem 65: 710-724.

26. Griffiths PD, Dobson BR, Jones GR, Clarke DT (1999) Iron in the basal ganglia in Parkinson's disease. An in vitro study using extended X-ray absorption fine structure and cryo-electron microscopy. Brain 122 ( Pt 4): 667-673.

27. Annanmaki T, Muuronen A, Murros K (2007) Low plasma uric acid level in Parkinson's disease. Mov Disord 22: 1133-1137.

28. Oakley AE, Collingwood JF, Dobson J, Love G, Perrott HR, et al. (2007) Individual dopaminergic neurons show raised iron levels in Parkinson disease. Neurology 68: 1820-1825.

29. Popescu BF, George MJ, Bergmann U, Garachtchenko AV, Kelly ME, et al. (2009) Mapping metals in Parkinson's and normal brain using rapid-scanning x-ray fluorescence. Phys Med Biol 54: 651-663.

30. Zhang J, Zhang Y, Wang J, Cai P, Luo C, et al. (2010) Characterizing iron deposition in Parkinson's disease using susceptibility-weighted imaging: an in vivo MR study. Brain Res 1330: 124-130.

31. Lotfipour AK, Wharton S, Schwarz ST, Gontu V, Schafer A, et al. (2012) High resolution magnetic susceptibility mapping of the substantia nigra in Parkinson's disease. J Magn Reson Imaging 35: 48-55.
