# Supplementary material for: Assessing Plasma Levels of Selenium, Copper, Iron and Zinc in Patients of Parkinson’s Disease
Source: PLoS One. 2013 Dec 10;8(12):e83060. doi: 10.1371/journal.pone.0083060 (PMC3858355; doi:10.1371/journal.pone.0083060)
Supplement: Table S2 — The graphite tube heating programs optimized for analyses of Se and Cu. (DOC) [file pone.0083060.s002.doc]

**Table S2 The graphite tube heating programs optimized for analyses of Se and Cu**

| Step | Se | | | Cu | | |
| --- | --- | --- | --- | --- | --- | --- |
|  | Temp (^o^C) | Ramp (s) | Hold (s) | Temp (^o^C) | Ramp (s) | Hold (s) |
| Drying | 85 | 5 | 0 | 85 | 5 | 0 |
| Drying | 95 | 25 | 0 | 95 | 15 | 0 |
| Drying | 130 | 5 | 10 | 130 | 5 | 10 |
| Ashing | 1000 | 10 | 15 | 1000 | 10 | 10 |
| Atomizing | 2250 | 0.8 | 3 | 2200 | 0.8 | 3 |
| Cleaning | 2600 | 2 | 0 | 2500 | 2 | 0 |
