# Supplementary material for: Assessing Plasma Levels of Selenium, Copper, Iron and Zinc in Patients of Parkinson’s Disease
Source: PLoS One. 2013 Dec 10;8(12):e83060. doi: 10.1371/journal.pone.0083060 (PMC3858355; doi:10.1371/journal.pone.0083060)
Supplement: Table S3 — Comparison of linear regression model and logistic regression model for the relationship between plasma element level and age in both PD and control subjects. (DOC) [file pone.0083060.s003.doc]

**Table S3** Comparison of linear regression model and logistic regression model for the relationship between plasma element level and age in both PD and control subjects

| **Element** | **Regression Method** |  | **Controls** | |  |  | **PD Patients** | |
| --- | --- | --- | --- | --- | --- | --- | --- | --- |
|  |  | R^2 | F | p value | | R^2 | F | p value |
| **Se** | Linear | 0.013 | 4.104 | 0.117 | | 0.009 | 2.155 | 0.229 |
|  | Logistic | 0.110 | 3.480 | 0.126 | | 0.005 | 1.245 | 0.328 |
| **Cu** | Linear | 0.005 | 1.648 | 0.267 | | 0 | 0.003 | 0.959 |
|  | Logistic | 0.005 | 1.57 | 0.328 | | 0 | 0.007 | 0.932 |
| **Fe** | Linear | 0.011 | 3.472 | 0.126 | | 0.029 | 6.937 | 0.036 |
|  | Logistic | 0.031 | 4.033 | 0.123 | | 0.022 | 5.313 | 0.088 |
| **Zn** | Linear | 0.001 | 0.431 | 0.585 | | 0.029 | 6.954 | 0.036 |
|  | Logistic | 0.004 | 1.140 | 0.328 | | 0.029 | 7.083 | 0.064 |
