# Supplementary material for: Assessing Plasma Levels of Selenium, Copper, Iron and Zinc in Patients of Parkinson’s Disease
Source: PLoS One. 2013 Dec 10;8(12):e83060. doi: 10.1371/journal.pone.0083060 (PMC3858355; doi:10.1371/journal.pone.0083060)
Supplement: Table S4 — Association analysis of plasma trace elements with PD by gender or age group. (DOC) [file pone.0083060.s004.doc]

**Table S4** Association analysis of plasma trace elements with PD by gender or age group^1^

| Subjects | Element | Category | Nagelkerke R square | First quartile | Second quartile | Third quartile | Fourth quartile | p value for trend^2^ |
| --- | --- | --- | --- | --- | --- | --- | --- | --- |
| Male | Se | Concentration | - | <78 | 78-102 | 103-126 | >126 | - |
|  |  | n (control/PD) | - | 40/25 | 57/36 | 30/20 | 26/40 | - |
|  |  | Model 1 | 0.048 | 0.389 (0.191,0.794) | 0.393(0.204,0.760) | 0.414 (0.193,0.888) | 1 | 0.217 |
|  |  | Model 2 | 0.415 | 0.249 (0.100,0.621) | 0.319 (0.143,0.711) | 0.374 (0.146,0.963) | 1 | 0.084 |
|  | Cu | Concentration | - | <819.75 | 819.75-990.5 | 990.6-1156.25 | >1156.25 | - |
|  |  | n (control/PD) | - | 33/35 | 32/37 | 46/23 | 42/26 | - |
|  |  | Model 1 | 0.040 | 1.716 (0.867,3.394) | 1.870 (0.947,3.695) | 0.806 (0.400,1.622) | 1 | 0.296 |
|  |  | Model 2 | 0.415 | 1.342 (0.562,3.204) | 1.719 (0.749,3.947) | 0.648 (0.268,1.564) | 1 | 0.519 |
|  | Fe | Concentration | - | <1113 | 1113-1444 | 1445-1928.25 | >1928.25 | - |
|  |  | n (control/PD) | - | 40/26 | 46/25 | 35/34 | 32/36 | - |
|  |  | Model 1 | 0.030 | 0.538 (0.262,1.101) | 0.458 (0.227,0.921) | 0.828 (0.419,1.637) | 1 | 0.217 |
|  |  | Model 2 | 0.415 | 0.298 (0.121,0.732) | 0.305 (0.128,0.729) | 0.443 (0.193,1.021) | 1 | 0.084 |
|  | Zn | Concentration | - | <825 | 825-1100 | 1101-1356.25 | >1356.25 | - |
|  |  | n (control/PD) | - | 10/55 | 43/36 | 45/17 | 55/13 | - |
|  |  | Model 1 | 0.311 | 23.428 (9.466,57.985) | 3.547 (1.676,7.506) | 1.579 (0.692,3.602) | 1 | 0.217 |
|  |  | Model 2 | 0.415 | 35.401 (13.012,96.313) | 4.961 (2.178,11.299) | 1.932 (0.782,4.775) | 1 | 0.084 |
| Female | Se | Concentration | - | <86.75 | 86.75-110.5 | 110.6-135 | >135 | - |
|  |  | n (control/PD) | - | 38/28 | 43/24 | 36/33 | 32/32 | - |
|  |  | Model 1 | 0.024 | 0.724 (0.362,1.450) | 0.569(0.282,1.149) | 0.912 (0.416,1.804) | 1 | 0.268 |
|  |  | Model 2 | 0.399 | 0.328 (0.131,0.825) | 0.311 (0.127,0.761) | 0.905 (0.391,2.094) | 1 | 0.115 |
|  | Cu | Concentration | - | <914 | 914-1072 | 1073-1241.75 | >1241.75 | - |
|  |  | n (control/PD) | - | 32/34 | 34/33 | 38/29 | 45/21 | - |
|  |  | Model 1 | 0.044 | 2.405 (1.176,4.918) | 2.139 (1.052,4.348) | 1.708 (0.837,3.488) | 1 | 0.068 |
|  |  | Model 2 | 0.399 | 1.291 (0.530,3.147) | 1.126 (0.479,2.644) | 1.177 (0.501,2.765) | 1 | 0.115 |
|  | Fe | Concentration | - | <997 | 997-1356.5 | 1356.6-1841 | >1841 | - |
|  |  | n (control/PD) | - | 44/22 | 36/31 | 38/29 | 31/35 | - |
|  |  | Model 1 | 0.037 | 0.435 (0.214,0.882) | 0.731 (0.368,1.453) | 0.657 (0.330,1.306) | 1 | 0.109 |
|  |  | Model 2 | 0.399 | 0.284 (0.115,0.697) | 0.648 (0.284,1.482) | 0.951 (0.418,2.167) | 1 | 0.115 |
|  | Zn | Concentration | - | <850 | 850-1075 | 1076-1375.75 | >1375.75 | - |
|  |  | n (control/PD) | - | 12/53 | 37/32 | 43/23 | 57/9 | - |
|  |  | Model 1 | 0.310 | 27.366(10.651,70.713) | 5.599 (2.390,13.115) | 3.376 (1.419,8.033) | 1 | 0.056 |
|  |  | Model 2 | 0.399 | 58.122 (19.247,175.517) | 7.535 (2.972,19.100) | 4.468 (1.772,11.268) | 1 | 0.08 |
| Age ≤55 | Se | Concentration | - | <80.5 | 80.5-108 | 109-135.5 | >135.5 | - |
|  |  | n (control/PD) | - | 17/10 | 20/9 | 17/9 | 17/10 | - |
|  |  | Model 1 | 0.015 | 0.935 (0.305,2.867) | 0.690(0.213,2.235) | 0.874 (0.281,2.721) | 1 | 0.664 |
|  |  | Model 2 | 0.270 | 0.635 (0.158,2.551) | 0.622 (0.146,2.656) | 0.646 (0.158,2.634) | 1 | 0.261 |
|  | Cu | Concentration | - | <856.5 | 865.5-1040 | 1041-1165.5 | >1165.5 | - |
|  |  | n (control/PD) | - | 18/9 | 13/15 | 21/6 | 19/8 | - |
|  |  | Model 1 | 0.085 | 1.178 (0.370,3.746) | 2.759 (0.905,8.408) | 0.705 (0.204,2.444) | 1 | 0.664 |
|  |  | Model 2 | 0.270 | 1.207 (0.294 ,4.963) | 2.580 (0.662,10.053) | 1.226 (0.292,5.150) | 1 | 0.59 |
|  | Fe | Concentration | - | <1031.5 | 1031.5-1475 | 1476-2075.5 | >2075.5 | - |
|  |  | n (control/PD) | - | 20/7 | 24/4 | 16/11 | 11/16 | - |
|  |  | Model 1 | 0.169 | 0.240 (0.074,0.778) | 0.112 (0.029,0.428) | 0.472 (0.158,1.414) | 1 | 0.336 |
|  |  | Model 2 | 0.270 | 0.188 (0.046,0.775) | 0.094 (0.019,0.457) | 0.336 (0.097,1.157) | 1 | 0.261 |
|  | Zn | Concentration | - | <900 | 900-1125 | 1126-1364 | >1364 | - |
|  |  | n (control/PD) | - | 14/12 | 18/12 | 17/9 | 22/5 | - |
|  |  | Model 1 | 0.074 | 3.768 (1.082,13.121) | 3.047 (0.873,10.640) | 2.223 (0.622,7.946) | 1 | 0.096 |
|  |  | Model 2 | 0.270 | 6.032 (1.321,27.552) | 2.811 (0.634,12.463) | 3.545 (0.760,16.540) | 1 | 0.261 |
| Age 55~65 | Se | Concentration | - | <94 | 94-109 | 110-140.25 | >140.25 | - |
|  |  | n (control/PD) | - | 17/10 | 19/11 | 13/14 | 10/18 | - |
|  |  | Model 1 | 0.086 | 0.358 (0.118,1.091) | 0.333(0.113,0.980) | 0.623 (0.210,1.852) | 1 | 0.118 |
|  |  | Model 2 | 0.506 | 0.096 (0.019,0.486) | 0.108 (0.025,0.476) | 0.415 (0.092,1.870) | 1 | 0.086 |
|  | Cu | Concentration | - | <862.75 | 862.75-1042.5 | 1042.6-1225 | >1225 | - |
|  |  | n (control/PD) | - | 9/19 | 16/12 | 18/10 | 16/12 | - |
|  |  | Model 1 | 0.115 | 3.369 (1.055,10.760) | 1.007 (0.340,2.979) | 0.799 (0.261,2.441) | 1 | 0.315 |
|  |  | Model 2 | 0.506 | 2.306 (0.505,10.527) | 0.899 (0.215,3.757) | 0.634 (0.150,2.684) | 1 | 0.573 |
|  | Fe | Concentration | - | <1188 | 1188-1581.5 | 1581.6-2059.75 | >2059.75 | - |
|  |  | n (control/PD) | - | 17/10 | 15/14 | 13/15 | 14/14 | - |
|  |  | Model 1 | 0.050 | 0.517 (0.167,1.597) | 0.947 (0.329,2.727) | 1.095 (0.374,3.208) | 1 | 0.315 |
|  |  | Model 2 | 0.506 | 0.604 (0.119,3.074) | 1.963 (0.426,9.046) | 1.470 (0.348,6.214) | 1 | 0.847 |
|  | Zn | Concentration | - | <806.25 | 806.25-1050 | 1051-1393.75 | >1393.75 | - |
|  |  | n (control/PD) | - | 5/23 | 13/17 | 16/10 | 25/3 | - |
|  |  | Model 1 | 0.361 | 38.983 (8.366,183.833) | 10.325 (2.531,42.120) | 5.485 (1.284,23.422) | 1 | 0.004 |
|  |  | Model 2 | 0.506 | 64.696 (9.909,422.409) | 26.818 (4.770,150.772) | 9.717 (1.688,55.933) | 1 | 0.044 |
| Age ≥65 | Se | Concentration | - | <80 | 80-105 | 106-127 | >127 | - |
|  |  | n (control/PD) | - | 48/28 | 49/39 | 40/37 | 35/43 | - |
|  |  | Model 1 | 0.026 | 0.474 (0.246,0.914) | 0.643(0.345,1.198) | 0.748 (0.395,1.413) | 1 | 0.024 |
|  |  | Model 2 | 0.470 | 0.206 (0.085,0.502) | 0.449 (0.200,1.008) | 0.535 (0.233,1.224) | 1 | 0.071 |
|  | Cu | Concentration | - | <864 | 864-1031 | 1032-1200 | >1200 | - |
|  |  | n (control/PD) | - | 36/43 | 37/44 | 48/32 | 51/28 | - |
|  |  | Model 1 | 0.043 | 2.176 (1.134,4.176) | 2.292 (1.199,4.379) | 1.218 (0.640,2.319) | 1 | 0.140 |
|  |  | Model 2 | 0.470 | 1.781 (0.743,4.269) | 1.995 (0.866,4.593) | 1.082 (0.475,2.464) | 1 | 0.168 |
|  | Fe | Concentration | - | <1013 | 1013-1350 | 1351-1775 | >1775 | - |
|  |  | n (control/PD) | - | 43/35 | 52/30 | 45/35 | 32/47 | - |
|  |  | Model 1 | 0.040 | 0.564 (0.299,1.065) | 0.398 (0.211,0.753) | 0.528 (0.281,0.992) | 1 | 0.231 |
|  |  | Model 2 | 0.470 | 0.248 (0.106,0.581) | 0.311 (0.141,0.686) | 0.315 (0.141,0.701) | 1 | 0.071 |
|  | Zn | Concentration | - | <821 | 821-1100 | 1101-1375 | >1375 | - |
|  |  | n (control/PD) | - | 9/70 | 44/43 | 59/21 | 60/13 | - |
|  |  | Model 1 | 0.380 | 40.852 (15.945,104.611) | 4.863 (2.307,10.253) | 1.766 (0.803,3.886) | 1 | 0.110 |
|  |  | Model 2 | 0.470 | 64.173 (22.358,184.191) | 7.121 (3.107,16.321) | 2.008 (0.844,4.777) | 1 | 0.071 |

^1^Odds ratios (95% CI) from logistic models were represented for dichoutomous outcomes (presence of PD) with the highest quartile as the reference group. Model 1 was for individual trace element adjusted with age and sex; Model 2 was for all four elements as ordinal variables adjusted with age and sex.

^2^Trend analyses across quartiles were performed using the ordinal variables containing median element concentrations for each quartile.
