# Supplementary material for: Assessing Plasma Levels of Selenium, Copper, Iron and Zinc in Patients of Parkinson’s Disease
Source: PLoS One. 2013 Dec 10;8(12):e83060. doi: 10.1371/journal.pone.0083060 (PMC3858355; doi:10.1371/journal.pone.0083060)
Supplement: Table S5 — Spearman correlation coefficients between plasma element concentrations and UPDRS scores in PD patients. (DOC) [file pone.0083060.s005.doc]

**Table S5** Spearman correlation coefficients between plasma element concentrations and UPDRS scores in PD patients

| Category | Se-UII | Se-UIII | Se-TU | Cu-UII | Cu-UIII | Cu-TU | Fe-UII | Fe-UIII | Fe-TU | Zn-UII | Zn-UIII | Zn-TU |
| --- | --- | --- | --- | --- | --- | --- | --- | --- | --- | --- | --- | --- |
| Total | -0.035 | -0.127 | -0.035 | -0.057 | -0.125 | -0.093 | -0.095 | -0.085 | 0.020 | **-0.199** | **-0.161** | -0.070 |
| Male | 0.128 | 0,058 | 0.135 | 0.087 | 0.050 | 0.164 | -0.169 | -0.183 | -0.045 | -0.201 | -0.081 | 0.025 |
| Female | -0.183 | **-0.283** | -0.164 | -0.199 | **0.268** | **-0.304** | -0.021 | -0.008 | 0.046 | -0.196 | **-0.238** | -0.164 |
| Age ≤55 | 0.199 | -0.051 | -0.022 | -0.200 | -0.372 | -0.323 | -0.101 | -0.032 | -0.027 | -0.235 | -0.382 | -0.405 |
| Age 55~65 | -0.185 | -0.219 | 0.032 | -0.194 | -0.150 | -0.053 | -0.198 | -0.173 | -0.080 | -0.329 | -0.357 | -0.224 |
| Age ≥65 | -0.047 | -0.097 | -0.053 | 0.028 | -0.058 | -0.061 | -0.037 | -0.067 | 0.072 | -0.116 | -0.046 | 0.052 |
| tremor-dominant | 0 | -0.249 | -0.125 | -0.759 | -0.769 | -0.637 | -0.024 | -0.370 | -0.259 | 0.024 | -0.312 | -0.210 |
| akinetic-rigid | -0.050 | -0.127 | -0.033 | -0.023 | -0.077 | -0.046 | -0.048 | -0.215 | 0.046 | -0.160 | 0.230 | -0.055 |
| mixed type | 0.012 | -0.124 | -0.024 | -0.023 | -0.122 | -0.095 | -0.159 | 0.033 | 0.099 | -0.246 | -0.103 | -0.072 |

Bold values are significant for p<0.05. UII, UPDRS II; UIII, UPDRS III; TU, total UPDRS.
