# Supplementary figures and images for: Assessing Plasma Levels of Selenium, Copper, Iron and Zinc in Patients of Parkinson’s Disease
Source: PLoS One. 2013 Dec 10;8(12):e83060. doi: 10.1371/journal.pone.0083060 (PMC3858355; doi:10.1371/journal.pone.0083060)

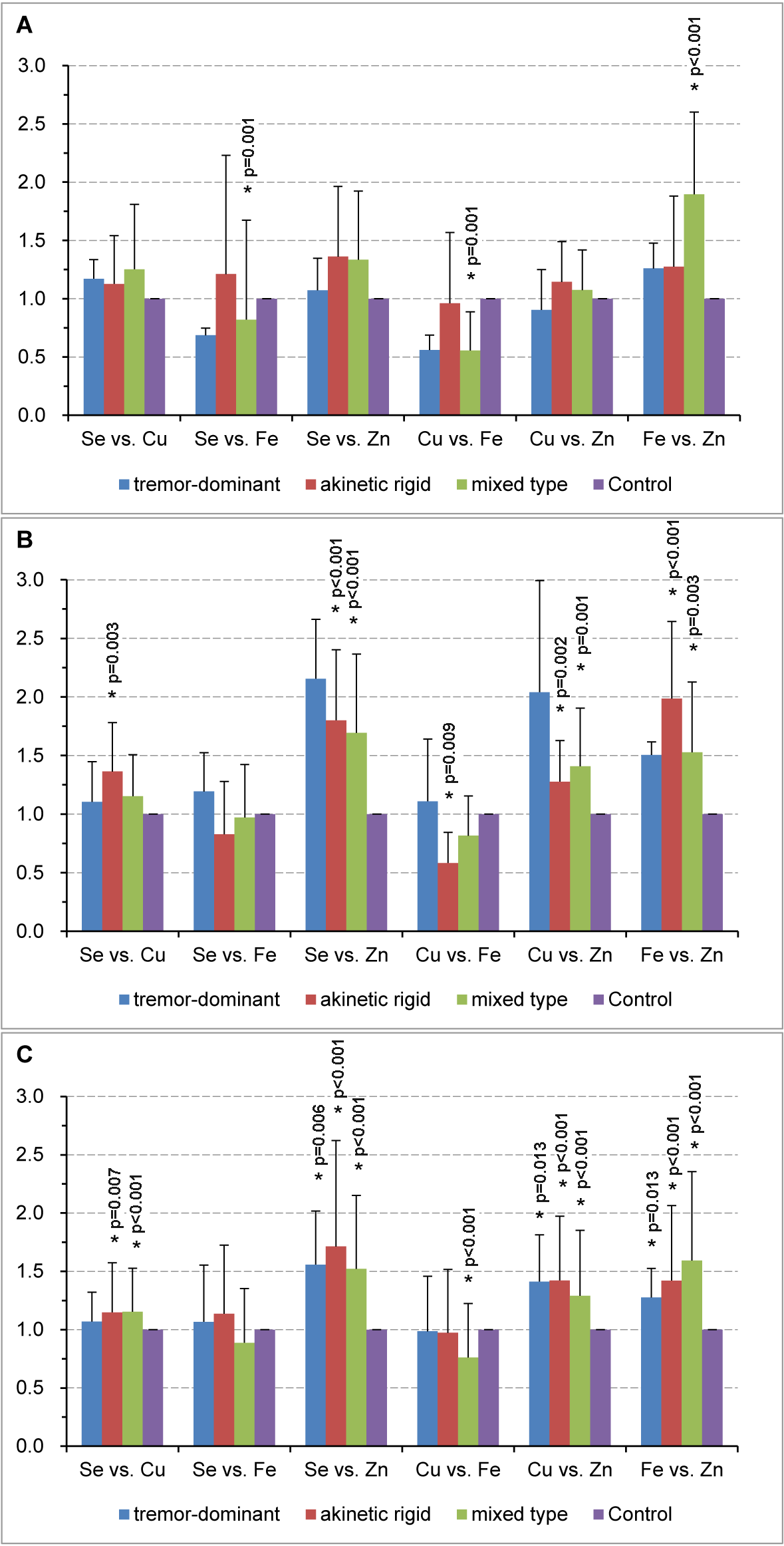

Supplement: Figure S1 — Element-element ratios for clinical subtypes based on age. Element-element ratio for each clinical subtype in PD patients was compared with age-matched controls of age ≤55 (A), 55~65 (B) and ≥65 (C). Histograms represent the fold changes of each element-element ratio compared to the mean value of their respecitve age-matched controls which was set as 1. Data were analyzed with Mann-Whitney U-test and significant difference was marked with * for p <0.05. (TIF) [file pone.0083060.s006.tif]

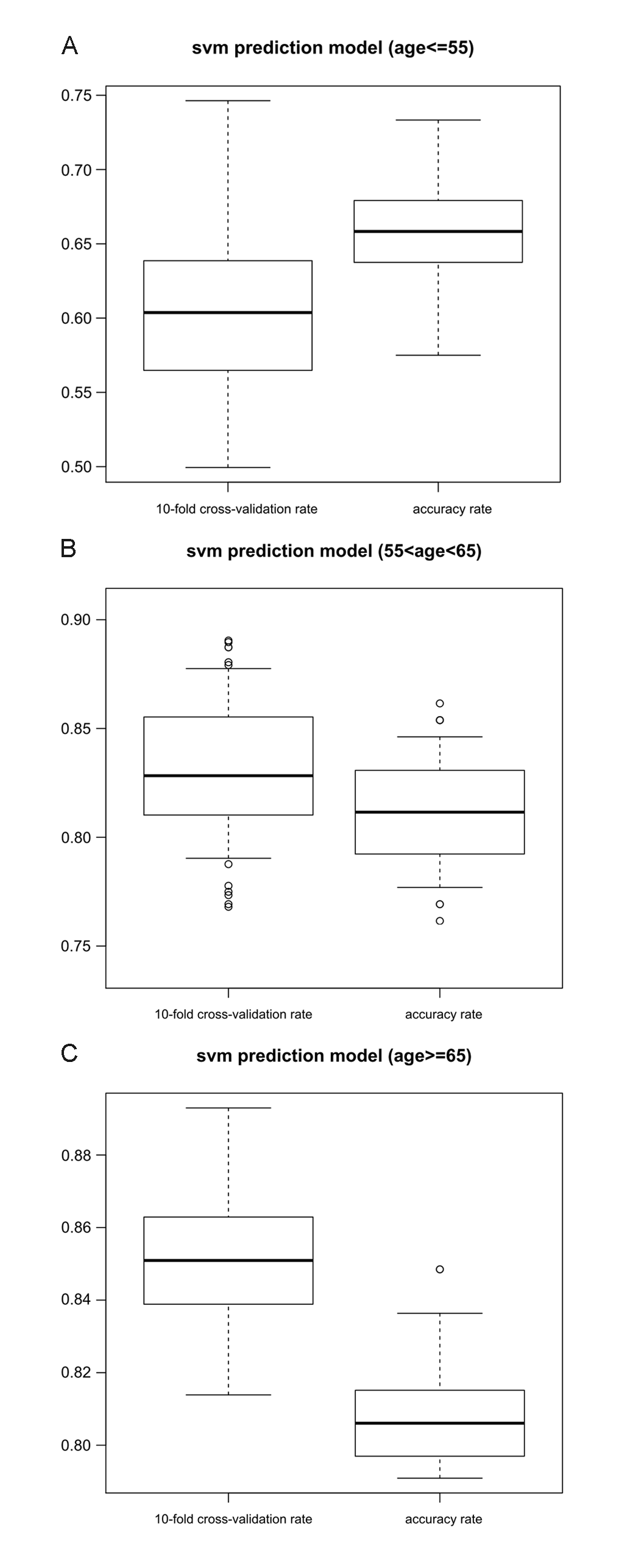

Supplement: Figure S2 — Generalized performance for SVM model based on ages. 10-fold cross-validation SVM model for PD prediction was built for 100 times based on age ≤55 (A), 55~65 (B) and ≥65 (C). (TIF) [file pone.0083060.s007.tif]
